# Newton Recursion Based Random Data-Reusing Generalized Maximum Correntropy Criterion Adaptive Filtering Algorithm

**DOI:** 10.3390/e24121845

**Published:** 2022-12-18

**Authors:** Ji Zhao, Yuzong Mu, Yanping Qiao, Qiang Li

**Affiliations:** 1 School of Information Engineering, Southwest University of Science and Technology, Mianyang 621010, China; 2 School of Power and Energy, Northwestern Polytechnical University, Xi’an 710072, China; 3 Science and Technology on Altitude Simulation Laboratory, Mianyang 621700, China

**Keywords:** generalized maximum correntropy, adaptive filtering algorithm, Newton recursion, data-reusing, acoustic echo cancellation

## Abstract

For system identification under impulsive-noise environments, the gradient-based generalized maximum correntropy criterion (GB-GMCC) algorithm can achieve a desirable filtering performance. However, the gradient method only uses the information of the first-order derivative, and the corresponding stagnation point of the method can be a maximum point, a minimum point or a saddle point, and thus the gradient method may not always be a good selection. Furthermore, GB-GMCC merely uses the current input signal to update the weight vector; facing the highly correlated input signal, the convergence rate of GB-GMCC will be dramatically damaged. To overcome these problems, based on the Newton recursion method and the data-reusing method, this paper proposes a robust adaptive filtering algorithm, which is called the Newton recursion-based data-reusing GMCC (NR-DR-GMCC). On the one hand, based on the Newton recursion method, NR-DR-GMCC can use the information of the second-order derivative to update the weight vector. On the other hand, by using the data-reusing method, our proposal uses the information of the latest *M* input vectors to improve the convergence performance of GB-GMCC. In addition, to further enhance the filtering performance of NR-DR-GMCC, a random strategy can be used to extract more information from the past *M* input vectors, and thus we obtain an enhanced NR-DR-GMCC algorithm, which is called the Newton recursion-based random data-reusing GMCC (NR-RDR-GMCC) algorithm. Compared with existing algorithms, simulation results under system identification and acoustic echo cancellation are conducted and validate that NR-RDR-GMCC can provide a better filtering performance in terms of filtering accuracy and convergence rate.

## 1. Introduction

Adaptive filters have been widely used in different engineering fields. Typical applications include system identification, active noise control, acoustic echo cancellation and channel equalization [1,2,3,4]. The adaptive filtering algorithms based on a mean square error (MSE) criterion mainly include the family of least mean square (LMS) algorithm, the tribe of affine projection (AP) algorithm and the class of least square algorithm [5,6,7]. Among them, the LMS algorithm is widely used because of its simple structure, low computational complexity, and good convergence in smooth environments. However, the above-mentioned MSE-based algorithms will have obvious filtering performance degradation in a non-Gaussian environment, especially in a noise environment with heavy-tailed distribution in which the typical heavy-tailed distribution noises include Laplace noise, Cauchy noise, mixed Gaussian noise, and alpha-stable distribution (α-SD) noise [8].

In order to enhance the ability of an adaptive filtering algorithm to suppress impulsive noise and improve the robustness of algorithms, from the perspective of similarity measurement, a series of nonlinear optimization criteria and/or cost functions have been proposed and applied to signal processing. Typical examples include the $l_{p}$-norm with p∈[1,2) [9], the *M*-estimation theory [10], and the information theoretic learning (ITL) family [11]. More details about robust adaptive signal processing schemes can be seen in a review article [12]. In the family of ITL criteria, thanks to all the even-order moment information of the error signal contained in the minimization of error entropy (MEE) [13] and the maximum correntropy criterion (MCC) [14,15], they are widely used in robust signal processing and machine learning. Generally speaking, the MCC criterion has a smaller computational burden than that of the MEE criterion. Although correntropy can provide a generalized similarity measure between two random variables, the Gaussian kernel function used in the MCC criterion may not always be the best choice. Therefore, as an extension of the MCC criterion, the correntropy criterion based on the generalized Gaussian density function, usually called the generalized maximum correntropy criterion (GMCC), has been proposed and widely used in the field of robust adaptive filtering [16,17,18,19,20,21]. Similar to the LMS algorithm family, when the correlation degree of the input signal gradually increases, the gradient-based generalized maximum correntropy criterion algorithm (GB-GMCC) will have obvious convergence performance degradation. In addition, the gradient method can only provide relevant first-order derivative information, which leads to certain deficiencies in the filtering performance of corresponding algorithms.

The GB-GMCC algorithm has the disadvantage of a slow convergence rate in highly colored input situations. In other words, the gradient method is not suitable for the correlated inputs. There are many methods to overcome this issue. Examples include affine projection, the recursive method [10], the subband method [22] and the data-reusing method, etc. However, relatively speaking, the complexity of the data-reusing method is usually lower. Therefore, we can consider it as a candidate method for solving the problem introduced by the highly colored inputs. On the other hand, the first-order derivative information may damage the convergence rate of GB-GMCC, therefore we can consider the second-order derivative information to remedy such issues, that is to say, the Newton recursion method can be a good choice to replace the gradient method.

A new robust adaptive filtering algorithm is proposed by the methods of data-reusing, Newton recursion and GMCC. The new algorithm is called the Newton recursion-based data-reusing generalized maximum correntropy criterion algorithm (NR-DR-GMCC). In addition, to further improve the convergence rate and filtering accuracy of the NR-DR-GMCC algorithm, based on the random data-reusing strategy, an enhanced version of NR-DR-GMCC is derived and is named the Newton recursion-based random data-reusing generalized maximum correntropy criterion algorithm (NR-RDR-GMCC). Some simulation results under system identification and acoustic echo cancellation are conducted and demonstrate that NR-RDR-GMCC can achieve a better filtering accuracy and faster convergence rate than existing related algorithms in the α-SD noise environment. The main contributions of this work are as follows:

- Based on the data-reusing and Newton recursion method, we propose the NR-DR-GMCC algorithm, which is derived by maximizing the generalized correntropy. Based on the data-reusing method, the information contained in the latest *M* input vectors can be obtained to combat the negative influence of highly colored inputs. Using the Newton recursion method, the second-order derivative information, i.e., the Hessian matrix, can be explored. Thus, the Hessian matrix can be used to update the weight vector leading to a faster convergence rate, thereby overcoming the disadvantage of the gradient method that only considers the first-order derivative information.
- Inspired by the random strategy method, NR-RDR-GMCC can be derived by introducing a cache window with length C≥M into the NR-RDR-GMCC algorithm. Compared with the NR-DR-GMCC algorithm, NR-RDR-GMCC has very similar computational complexity except for requiring extra memory to contain *C* input vectors. However, NR-RDR-GMCC can explore more historical information and avoid the effects of consecutive large outliers through the cache window and use it to update the weight, thereby improving the filtering performance of the NR-DR-GMCC algorithm.

The rest of this paper is organized as follows. In Section 2, some preliminaries about α-SD Noise, the GMCC criterion, and the Newton recursion method are reviewed. Section 3 presents our proposed algorithms, namely, NR-DR-GMCC and NR-RDR-GMCC. Simulation results are presented in Section 4. Finally, the conclusion is given in Section 5.

## 2. Preliminaries

### 2.1. Alpha-Stable Distribution Noise

In this paper, the impulsive noise can be simulated by α-SD, and the noise model can construct the ubiquitous non-Gaussian noise with strong impulsive characteristics well [23]. The characteristic function of this model is defined as:

(1) $$\Phi \left(\theta \right)=\left\{\begin{array}{cc} \exp\left\{j\delta \theta -{\lambda |\theta |}^{\alpha }\left[1+j\varsigma \mathrm{sgn}\left(\theta \right)\tan\frac{\alpha \pi }{2}\right]\right\}, & \alpha \neq 1\\ \exp\left\{j\delta \theta -\lambda |\theta |\left[1+j\varsigma \mathrm{sgn}\left(\theta \right)\frac{2}{\pi }\log\left|t\right|\right]\right\}, & \alpha =1 \end{array}\right.,$$

where $j^{2}=-1$; α∈(0,2] denotes a characteristic exponent measuring the thickness of the tail of a distribution. The smaller the value of α, the thicker the tail of the corresponding distribution, and the more significant the impulsive characteristics. When the value of α is close to 2, the corresponding distribution is close to the Gaussian distribution; λ>0 denotes a dispersion parameter, which is similar to the variance of a Gaussian distribution; ς∈[−1,1] means the symmetry parameter, which can define the inclination of the distribution; and δ∈(−∞,+∞) denotes the location parameter. In general, when α=2, ς=0, α-SD is equivalent to a Gaussian distribution. In this paper, for the sake of simplicity, the parameter vector of α-SD is denoted as $d_{\alpha }^{T}=\left[\alpha ,\varsigma ,\delta ,\lambda \right]$.

### 2.2. Generalized Maximum Correntropy Criterion

Correntropy measures the local similarity between two random variables *X* and *Y* [11],

(2) $$C_{\sigma }\left(X,Y\right)=E\left[\kappa_{\sigma }\left(X,Y\right)\right]=\int \kappa_{\sigma }\left(x,y\right)dF_{X,Y}\left(x,y\right),$$

where E[·] stands for the expectation operator; $F_{X,Y}\left(x,y\right)$ denotes the joint distribution function of *X* and *Y*; and $\kappa_{\delta }\left(\cdot ,\cdot \right)$ denotes a Gaussian kernel defined as:

(3) $$\kappa_{\sigma }\left(x,y\right)=\exp\left({-\sigma |x-y|}^{2}\right),$$

where σ>0 means a kernel factor. In practice, the joint distribution $F_{X,Y}\left(x,y\right)$ is usually unknown, and thus only a finite number of data ${\left\{x_{n},y_{n}\right\}}_{n=1}^{N}$ are used to derive the sample estimator of (3) as

(4) $${\hat{C}}_{\sigma }\left(X,Y\right)=\frac{1}{N}\sum_{n=1}^{N}\kappa_{\sigma }\left(x_{n},y_{n}\right).$$

From a probabilistic statistical point of view, the maximization of correntropy can lead to the error producing the maximum probability density at the origin, and thus the Equation (4) can be regarded as a nonlinear cost function, and it has been widely adopted in the field of robust adaptive signal processing. However, the Gaussian kernel function used in the estimation (4) may not always be the best choice. Therefore, other types of kernel functions can be used to replace the Gaussian kernel, such as a kernel function based on the generalized Gaussian density (GGD) function, i.e.,

(5) $$\kappa_{s,\tau }\left(x,y\right)=\gamma_{s,t}\exp\left({-\tau |x-y|}^{s}\right),$$

where $\tau =t^{-s}$ is the kernel parameter; *s* and *t* are positive numbers and present the shape parameter and the scale factor, respectively; and $\gamma_{s,t}=s/2t\Gamma \left(s^{-1}\right)$ denotes the normalization constant with Γ(·) being the Gamma function. Injecting GGD into (4), a new sample estimator can be obtained as

(6) $${\hat{C}}_{s,\tau }\left(X,Y\right)=\frac{1}{N}\sum_{n=1}^{N}\kappa_{s,\tau }\left(x_{n},y_{n}\right).$$

In the field of robust adaptive signal processing, (6) is often referred to as generalized correntropy. In applications such as regression analysis and classification studies, the correntropy loss (C-loss) metric can be used in place of correntropy [24]. Inspired by this, the generalized C-loss (GC-loss) function between the random variables *X* and *Y* can be defined as

(7) $$J\left(X,Y\right)={\hat{C}}_{s,\tau }\left(0\right)-\frac{1}{N}\sum_{n=1}^{N}\kappa_{s,\tau }\left(e_{n}\right),$$

where $e_{n}=x_{n}-y_{n}$ means the error information. Equation (7) shows that minimizing GC-loss is equivalent to maximizing generalized correntropy, which is usually denoted as GMCC and is widely used in the field of adaptive filtering [18,20,25].

### 2.3. Newton Recursion

Using the second-order Taylor formula to expand a continuous function f(x) at $x_{0}$,

(8) $$f\left(x\right)\approx f\left(x_{0}\right)+\left(x-x_{0}\right)g\left(x_{0}\right)+\frac{1}{2}\left(x-x_{0}\right)H\left(x_{0}\right),$$

where $g(x_{0})$ represents the gradient of f(x) at $x_{0}$ and $H(x_{0})$ is the gradient of $g(x_{0})$ at $x_{0}$. Therefore, based on (8), we can obtain the gradient of f(x) with respect to *x* as

(9) $$g\left(x\right)\approx g\left(x_{0}\right)+H\left(x_{0}\right)\left(x-x_{0}\right).$$

Setting g(x)=0, we obtain

(10) $$x\approx x_{0}-H{\left(x_{0}\right)}^{-1}g\left(x_{0}\right),$$

which leads to the general form of the Newton recursion method [26],

(11) $$x_{k+1}=x_{k}-H{\left(x_{k}\right)}^{-1}g\left(x_{k}\right).$$

This recursive method is often used to obtain approximate solutions of nonlinear equations g(x)=0. If g(x) is a real-valued function, then $x_{k+1}$ represents the point where the tangent $y-g\left(x_{k}\right)=H\left(x_{k}\right)\left(x-x_{k}\right)$ of the function g(x) at point $x_{k}$ intersects the *x*-axis [27]. It is worth noting that when g(x) is a vector function, the H(x) becomes a Hessian matrix and, in this case, the use of Newton recursion must ensure that the Hessian matrix is positive definite.

## 3. The Proposed Algorithms

### 3.1. GB-GMCC

The following linear model is considered to reconstruct an unknown system such that the output y(n) of a adaptive filter matches the desired signal d(n)

(12) $$d\left(n\right)=u{\left(n\right)}^{T}w^{o}+v\left(n\right),$$

where $u\left(n\right)={\left[u\left(n\right),u\left(n-1\right),\ldots ,u\left(n-L+1\right)\right]}^{T}$ denotes the input vector with length *L*; $w^{o}={\left[w_{1}^{o},w_{2}^{o},\ldots ,w_{L}^{o}\right]}^{T}$ means the intrinsic weight vector of the unknown system; and v(n) is the α-SD noise. For simplicity, the normalized constant $\gamma_{s,t}$ in the GGD function is ignored, and the sample estimator in (6) is replaced by an instantaneous estimator to establish the following cost function,

(13) $$J\left(w\right)=\exp\left(-\tau |e\left(n\right){|}^{s}\right),$$

where $e\left(n\right)=d\left(n\right)-u{\left(n\right)}^{T}w$ denotes the estimation error and $w={\left[w_{1},w_{2},\ldots ,w_{L}\right]}^{T}$ means the estimated weight vector. Calculate the gradient of (13), and use the gradient ascent method to obtain the weight vector update formula of the GB-GMCC algorithm as

(14) $$w\left(n+1\right)=w\left(n\right)+\mu f\left(e\left(n\right)\right)u\left(n\right),$$

where

(15) $$f\left(e\left(n\right)\right)=\exp\left(-\tau |e\left(n\right){|}^{s}\right){\left|e\left(n\right)\right|}^{s-2}e\left(n\right)$$

denotes a nonlinear function of the error information; and μ>0 stands for the learning step size.

### 3.2. NR-DR-GMCC

Observing from (14), one can find that the GB-GMCC algorithm only uses the current input vector u(n) to update the weight vector. Although this method has a lower computational complexity, it results in the slower convergence rate of the GB-GMCC algorithm when facing the increment of the correlation of the input signal. This problem can be overcome by using the data-reusing method, and thus the cost function in (13) can be changed as

(16) $$J\left(w\right)=\exp\left(-\tau {∥e\left(n\right)∥}_{s}^{s}\right),$$

where

(17) $$e\left(n\right)={\left[e\left(n\right),e\left(n-1\right),\ldots ,e\left(n-M+1\right)\right]}^{T}$$

represents an error vector consisting of *M* recent error signals and ${∥\cdot ∥}_{s}^{s}$ means the *s* power of $\ell_{s}$-norm. In addition, the GB-GMCC algorithm updates the weight vector by using the gradient ascent strategy, which only considers the first-order derivative information. When all elements in the gradient are zero values, the stationary point of the gradient ascent method may be a local maximum and/or a saddle point. In other words, the gradient ascent method is an inefficient or even ineffective optimization method in some situations. To this end, inspired by the Newton recursion strategy, we can derive a new generalized maximum correntropy criterion algorithm as follows.

Let $g\left(w\right)=\frac{\partial J\left(w\right)}{\partial w}$, and take the gradient of (16) with respect to w as

(18) $$g\left(w\right)=\frac{\partial J\left(w\right)}{\partial w}=\tau s\exp\left(-\tau {∥e\left(n\right)∥}_{s}^{s}\right)\sum_{i=n}^{n-M+1}{\left|e\left(i\right)\right|}^{s-2}e\left(i\right)u\left(i\right),$$

where $\mathrm{sgn}\left(\cdot \right)$ is a symbolic function. Further denoting $\frac{\partial g\left(w\right)}{\partial w}$ as $H\left(w\right)$, we can obtain the Hessian matrix as

(19) $$H\left(w\right)=\tau s\exp\left(-\tau {∥e\left(n\right)∥}_{s}^{s}\right)\left(\tau s{\left(\sum_{i=n}^{n-M+1}{\left|e\left(i\right)\right|}^{s-2}e\left(i\right)u\left(i\right)\right)}^{2}-\left(s-1\right)\sum_{i=n}^{n-M+1}u\left(i\right)u{\left(i\right)}^{T}\right).$$

Based on the following definitions, the $g\left(w\right)$ and $H\left(w\right)$ can be represented as more compact formations as

(20) $$\left\{\begin{array}{c} g\left(w\right)=\tau s\exp\left(-\tau {∥e\left(n\right)∥}_{s}^{s}\right)U\left(n\right)\varphi \left(n\right)\\ H\left(w\right)=\tau s\exp\left(-\tau {∥e\left(n\right)∥}_{s}^{s}\right)\left(\tau sU\left(n\right)\varphi \left(n\right)\varphi {\left(n\right)}^{T}U{\left(n\right)}^{T}-\left(s-1\right)U\left(n\right)U{\left(n\right)}^{T}\right)\\ U\left(n\right)=\left[u\left(n\right),u\left(n-1\right),\ldots ,u\left(n-M+1\right)\right]\\ \varphi \left(n\right)=b\left(n\right)⊙\mathrm{sgn}\left(e\left(n\right)\right)\\ b\left(n\right)={\left[{\left|e\left(n\right)\right|}^{s-1},{\left|e\left(n-1\right)\right|}^{s-1},\ldots ,{\left|e\left(n-M+1\right)\right|}^{s-1}\right]}^{T}\\ \mathrm{sgn}\left(e\left(n\right)\right)={\left[\mathrm{sgn}\left(e\left(n\right)\right),\mathrm{sgn}\left(e\left(n-1\right)\right),\ldots ,\mathrm{sgn}\left(e\left(n-M+1\right)\right)\right]}^{T} \end{array}\right.,$$

where ⊙ is the Hartman product. Therefore, based on Newton recursion in (11), a weight vector update formation of the *Newton recursion-based data-reusing generalized maximum correntropy criterion* (NR-DR-GMCC) algorithm is derived as

(21) $$w\left(n+1\right)=w\left(n\right)-\eta {\left(\tau sR_{u,\varphi }\left(n\right)-\left(s-1\right)R_{u}\left(n\right)+\epsilon I\right)}^{-1}U\left(n\right)\varphi \left(n\right),$$

where $R_{u,\varphi }\left(n\right)=U\left(n\right)\varphi \left(n\right)\varphi {\left(n\right)}^{T}U{\left(n\right)}^{T}$; $R_{u}=U\left(n\right)U{\left(n\right)}^{T}$ is a covariance matrix of the input matrix $U\left(n\right)$; η>0 is step-size; and ϵ>0 denotes a small-valued smoothing factor.

### 3.3. NR-RDR-GMCC

Although the filtering performance of NR-DR-GMCC can be improved by digging much information contained in the latest *M* input vectors, the computational complexity of the algorithm will be increased when the data-reusing order *M* is large. Therefore, to balance the computational burden and the good filtering performance, we further inject a random strategy of data-reusing into the NR-DR-GMCC algorithm, and thus an enhanced version of NR-DR-GMCC is obtained as follows.

Based on this, we can define the error vector as

(22) $$e_{r}\left(n\right)={\left[e\left(r_{n}^{\left(1\right)}\right),e\left(r_{n}^{\left(2\right)}\right),\ldots ,e\left(r_{n}^{\left(M\right)}\right)\right]}^{T},$$

which is composed of randomly selected error signals in which $i\in \left[1,M\right]$, $r_{n}^{\left(i\right)}$ denotes the index of an interval [n−C+1,n] and satisfies $a\neq b\to r_{n}^{\left(a\right)}\neq r_{n}^{\left(b\right)}$, where C>M is the length of a cache window. Then, (16) can be modified as

(23) $$J\left(w\right)=\exp\left(-\tau {∥e_{r}\left(n\right)∥}_{s}^{s}\right).$$

Based on some similar operations to (18) and (19), we can obtain a weight vector update formation of the *Newton recursion-based random data-reusing generalized maximum correntropy criterion* (NR-RDR-GMCC) algorithm as

(24) $$w\left(n+1\right)=w\left(n\right)-\eta {\left(\tau sR_{u,\varphi }^{r}\left(n\right)-\left(s-1\right)R_{u}^{r}\left(n\right)+\epsilon I\right)}^{-1}U_{r}\left(n\right)\varphi_{r}\left(n\right),$$

in which the following symbols are used

(25) $$\left\{\begin{array}{c} U_{r}\left(n\right)=\left[u\left(r_{n}^{\left(1\right)}\right),u\left(r_{n}^{\left(2\right)}\right),\ldots ,u\left(r_{n}^{\left(M\right)}\right)\right]\\ \varphi_{r}\left(n\right)=b_{r}\left(n\right)⊙\mathrm{sgn}\left(e_{r}\left(n\right)\right)\\ b_{r}\left(n\right)={\left[{\left|e\left(r_{n}^{\left(1\right)}\right)\right|}^{s-1},{\left|e\left(r_{n}^{\left(2\right)}\right)\right|}^{s-1},\ldots ,{\left|e\left(r_{n}^{\left(M\right)}\right)\right|}^{s-1}\right]}^{T}\\ \mathrm{sgn}\left(e_{r}\left(n\right)\right)={\left[\mathrm{sgn}\left(e\left(r_{n}^{\left(1\right)}\right)\right),\mathrm{sgn}\left(e\left(r_{n}^{\left(2\right)}\right)\right),\ldots ,\mathrm{sgn}\left(e\left(r_{n}^{\left(M\right)}\right)\right)\right]}^{T}\\ R_{u,\varphi }^{r}\left(n\right)=U_{r}\left(n\right)\varphi_{r}\left(n\right)\varphi_{r}{\left(n\right)}^{T}U_{r}{\left(n\right)}^{T}\\ R_{u}^{r}\left(n\right)=U_{r}\left(n\right)U_{r}{\left(n\right)}^{T} \end{array}\right..$$

**Figure 1:**
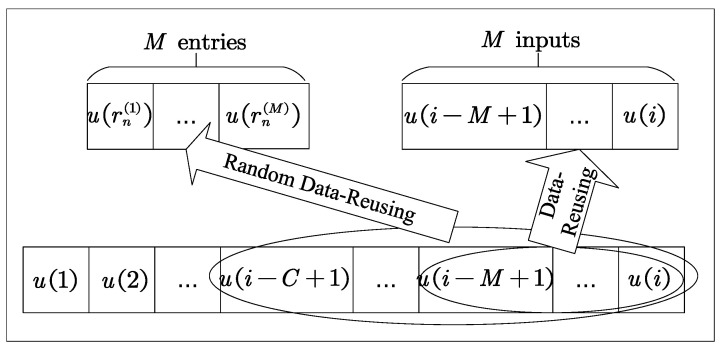
Schematic diagram of the data-reusing and the random data-reusing methods.

Remarks: (1) Based on the data-reusing method, the NR-DR-GMCC algorithm can use the latest *M* input information to update the weight equation, remitting the convergence rate degradation problem of GB-GMCC due to the increased correlation of the input signal; (2) In comparison with GB-GMCC only using the information of the first-order derivative, the NR-DR-GMCC algorithm can use the second-order information provided by the Newton recursion method, and thus NR-DR-GMCC achieves faster convergence behavior; (3) As an improved version of NR-DR-GMCC, the NR-RDR-GMCC algorithm uses a cache window with length C>M to store the latest *C* input vectors. Then, to update the weight vector, NR-RDR-GMCC randomly selects *M* input vectors from the cache window. According to the random data-reusing method, NR-RDR-GMCC obtains abundant error information from the cache window and avoids the convergence loss caused by continuous outliers. Furthermore, when the data-reusing order *M* is fixed, NR-RDR-GMCC does not have a similar computational complexity to that of NR-DR-GMCC with data-reusing order *M*, but also achieves a better filtering performance than NR-DR-GMCC. Moreover, observing from (12) and (16), one can find that, when C=M, the NR-RDR-GMCC algorithm becomes the NR-DR-GMCC algorithm. That is to say, NR-RDR-GMCC can be regarded as an effective extension of NR-DR-GMCC. Algorithm 1 summarizes the pseudocode of the proposed algorithms. Moreover, Figure 1 plots the schematic diagram of the data-reusing and the random data-reusing methods, and shows the difference between these two methods, namely, the data-reusing method only utilizes the latest *M* inputs, whereas the random data-reusing method saves the latest *C* inputs and randomly selects *M* entries from these *C* inputs.
**Algorithm 1.** Pseudocode of Proposed Algorithms1**Initialization**: $0<M<C,\epsilon >0,\eta >0,s>0,\tau >0,w\left(0\right)=0$2**Computation:**3 **for** n>0 **do**4  **if** n≤M5   $i=n,\;u\left(r_{n}^{\left(i\right)}\right)=u\left(i\right)$6  **else if** M<n≤C7    $r_{n}^{\left(i\right)}\in \left[1,n\right]\to u\left(r_{n}^{\left(i\right)}\right)$8  **else** n>C9    $r_{n}^{\left(i\right)}\in \left[n+C-1,n\right]\to u\left(r_{n}^{\left(i\right)}\right)$10  **end if**11  $U_{r}\left(n\right)=\left[u\left(r_{n}^{\left(1\right)}\right),u\left(r_{n}^{\left(2\right)}\right),\ldots ,u\left(r_{n}^{\left(M\right)}\right)\right]$12  $e_{r}\left(n\right)=d_{r}\left(n\right)-U_{r}{\left(n\right)}^{T}w\left(n\right)$13  $b_{r}\left(n\right)={\left[{\left|e\left(r_{n}^{\left(1\right)}\right)\right|}^{s-1},{\left|e\left(r_{n}^{\left(2\right)}\right)\right|}^{s-1},\ldots ,{\left|e\left(r_{n}^{\left(M\right)}\right)\right|}^{s-1}\right]}^{T}$14  $\mathrm{sgn}\left(e_{r}\left(n\right)\right)={\left[\mathrm{sgn}\left(e\left(r_{n}^{\left(1\right)}\right)\right),\mathrm{sgn}\left(e\left(r_{n}^{\left(2\right)}\right)\right),\ldots ,\mathrm{sgn}\left(e\left(r_{n}^{\left(M\right)}\right)\right)\right]}^{T}$15  $\varphi_{r}\left(n\right)=b_{r}\left(n\right)⊙\mathrm{sgn}\left(e_{r}\left(n\right)\right)$16  $H_{r}\left(w\left(n\right)\right)=\tau sU_{r}\left(n\right)\varphi_{r}\left(n\right)\varphi_{r}{\left(n\right)}^{T}U_{r}{\left(n\right)}^{T}-\left(s-1\right)U_{r}\left(n\right)U_{r}{\left(n\right)}^{T}$17  $g_{r}\left(w\left(n\right)\right)=U_{r}\left(n\right)\varphi_{r}\left(n\right)$18  $w\left(n+1\right)=w\left(n\right)-\eta {\left(H_{r}\left(w\left(n\right)\right)+\epsilon I\right)}^{-1}g_{r}\left(w\left(n\right)\right)$19**End for**

### 3.4. Computation Complexity Analysis

Different from the traditional GB-GMCC algorithm, the random strategy is applied in NR-RDR-GMCC for efficiently reusing the past *M* input vectors, and thus our proposal can extract more historical information supporting the weight vector update inspired by Newton recursion. Although, compared with GB-GMCC algorithm, our proposed NR-RDR-GMCC has a higher computational complexity, it is worthwhile since NR-RDR-GMCC outperforms GB-GMCC in terms of filtering accuracy as will be shown in the simulation results. It is worth noting that the matrix inverse involved in NR-RDR-GMCC can be achieved with the help of some iterative optimization methods, thereby reducing the computational complexity of the NR-RDR-GMCC algorithm [28]. In addition, Table 1 lists the computational complexity of the proposed NR-RDR-GMCC and other related algorithms per iteration in terms of multiplications and additions in which *M* represents the order of data-reusing and/or projection, *L* stands for the tap length, $N_{\exp}^{\tau ,s}$ indicates the computational complexity associated with the nonlinear function $\exp\left(-\tau {\left|e\left(i\right)\right|}^{s}\right){\left|e\left(i\right)\right|}^{s-1}$ [20], and $O(L^{3})$ is the computational burden required for the direct inverse of the square matrix in the L×L dimension.

## 4. Simulation Results

### 4.1. System Identification

In order to evaluate the filtering performance of the NR-RDR-GMCC algorithm, we consider the system identification problem represented in (12), where the unknown weight vector $w^{o}$ is randomly generated and the tap length L=32. Impulsive noise is modeled by α-SD with the parameter vector $d_{\alpha }^{T}=\left[1.5,0,0,0.1\right]$. The input signal u(n) is generated by a zero-mean Gaussian noise with unit variance filtered by the following second-order system:

(26) $$H\left(z\right)=\frac{1+0.5z^{-1}+0.81z^{-2}}{1-0.59z^{-1}+0.4z^{-2}}.$$

To compare the filtering performance of competing algorithms, the normalized mean square deviation (NMSD) is used, i.e.,

(27) $$\mathrm{NMSD}=20\mathrm{lg}\left(\frac{∥w-w^{o}∥}{∥w^{o}∥}\right);$$

in addition, all NMSD simulation results are averaged over 100 independent experiments.

**Figure 2:**
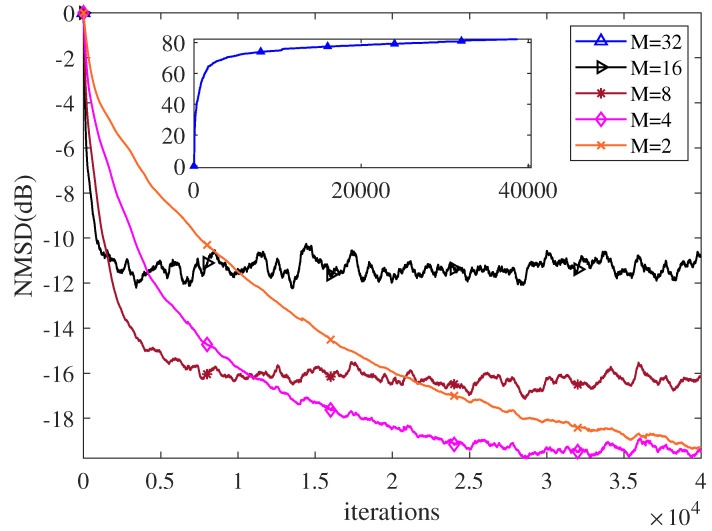
The NMSD curves of NR-RDR-GMCC with different values of $M\in \left\{2,4,6,8,16,32\right\}$.

Firstly, in order to study the effect of the order *M* of data-reusing on the convergence performance of the NR-RDR-GMCC algorithm, we set different *M* from $\in \left\{2,4,6,8,16,32\right\}$, and the other parameters are fixed as C=512, s=1.5, τ=0.0001, η=0.005, and ϵ=0.0001. Figure 2 plots the corresponding NMSD curves. From Figure 2, we can observe that: (1) When *M* is large, such as M=32, the NR-RDR-GMCC algorithm does not converge; (2) Under the premise of the convergence of NR-RDR-GMCC, by appropriately increasing the *M* value, such as from 2 to 16, the convergence rate of NR-RDR-GMCC will be significantly improved; (3) With some moderate values, such as M=4, NR-RDR-GMCC can achieve an acceptable convergence rate and the best filtering accuracy. Therefore, for NR-RDR-GMCC, in practical applications, it is necessary to select a proper value of *M* to make the trade-off among the computational complexity, filtering accuracy and convergence rate.

**Figure 3:**
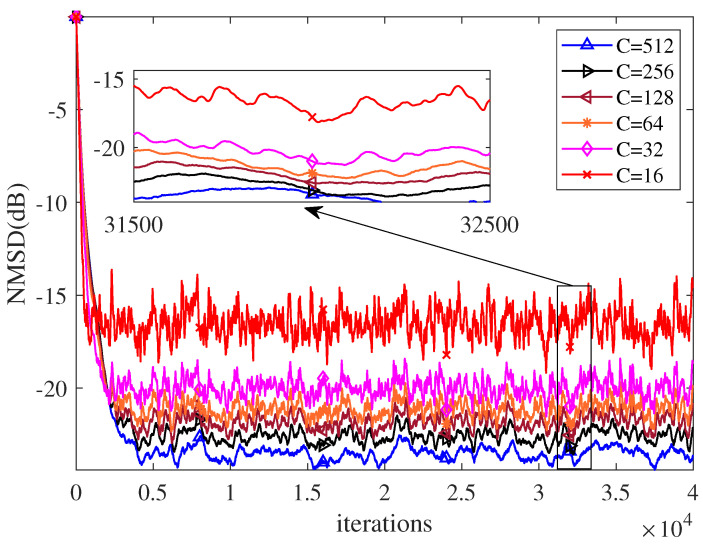
The NMSD curves of NR-RDR-GMCC with different values of $C\in \left\{16,32,64,128,256,512\right\}$.

Secondly, we investigate the influence of the length of cache window *C* on the performance of NR-RDR-GMCC. To this end, some values of *C* from $\left\{16,32,64,128,256,512\right\}$ are set and other parameters are fixed as M=16, s=1.5, τ=0.0001, η=0.005 and ϵ=0.0001. The corresponding NMSD curves are plotted in Figure 3, which clearly shows that: (1) The filtering accuracy of NR-RDR-GMCC can be enhanced by increasing the values of *C*. In addition, with some large *C* values, such as C∈{256,512}, the jitter behavior of NR-RDR-GMCC can be restrained, thereby improving the stability of our proposed algorithm; (2) When C=M=16, NR-RDR-GMCC reduces to the NR-DR-GMCC algorithm. However, compared to NR-RDR-GMCC with other large *C* values, the NR-DR-GMCC algorithm realizes the fastest convergence rate at the cost of the worst accuracy. That is to say, with some moderate *C* values, NR-RDR-GMCC can achieve a good balance between a smaller misadjustment and a good convergence rate.

**Figure 4:**
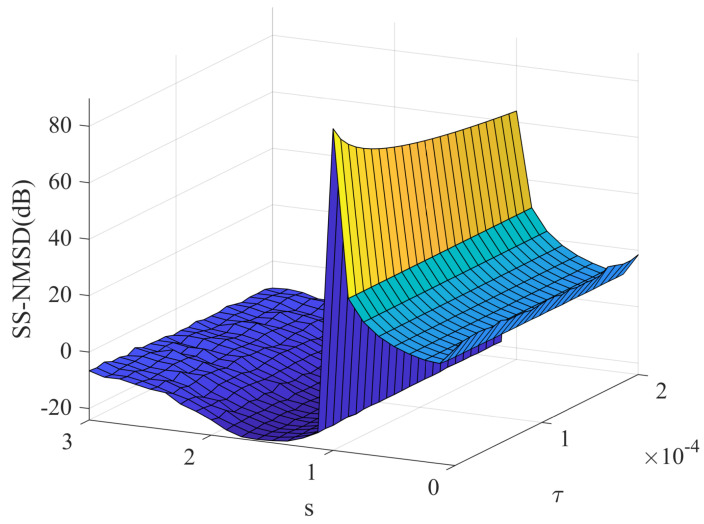
The steady-state NMSD results of NR-RDR-GMCC with different values of s∈(0,3) and τ∈(0.00001,0.0002).

**Figure 5:**
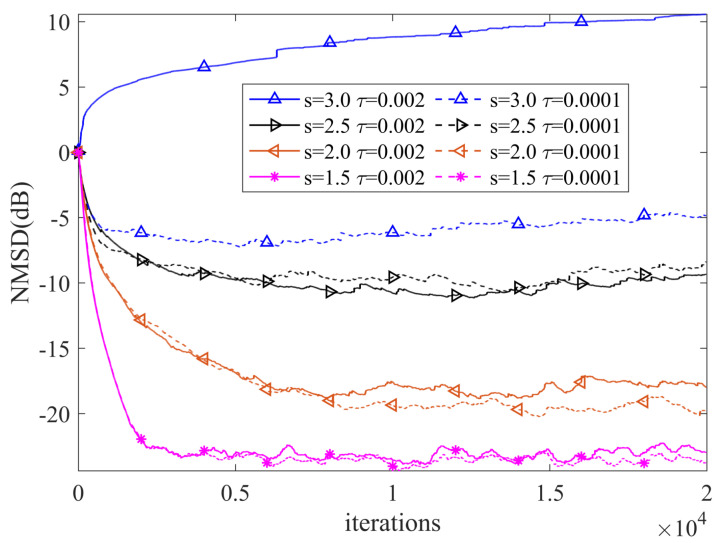
The NMSD curves of NR-RDR-GMCC with different values of $\tau \in \left\{0.00001,0.002\right\}$ and $s\in \left\{1.5,2.0,2.5,3.0\right\}$.

Thirdly, we study the effect of the shape parameter *s* and kernel parameter τ on the filtering accuracy of NR-RDR-GMCC. To this end, different *s* and τ values are considered and other parameters are set as C=512, M=16, η=0.005 and ϵ=0.0001. Figure 4 shows the steady-state NMSD (SS-NMSD) estimated by the last 100 NMSD for NR-RDR-GMCC with respect to various values of s∈(0,3) and τ∈(0.00001,0.0002). From this figure we can observe that: (1) With the same shape parameter *s*, different τ values lead to NR-RDR-GMCC obtaining very similar filtering accuracy; (2) With the same kernel parameter τ, different *s* values result in obvious steady-state behaviors of NR-RDR-GMCC. That is to say, when s∈(1.0,3.0), the filtering accuracy will gradually increase with the decrease of *s*; when s=1.0, the algorithm mutates into a divergent state; when s∈(0.0,1.0), the algorithm does not converge. In addition, to clearly show the filtering behavior, some NMSD curves of NR-RDR-GMCC with $\tau \in \left\{0.00001,0.002\right\}$ and $s\in \left\{1.5,2.0,2.5,3.0\right\}$ are plotted in Figure 5. From this figure, we have the following observations: (1) When the *s* parameter is same, in most cases, the NR-RDR-GMCC algorithms with τ=0.00001 or τ=0.002 realize similar convergence behavior; (2) With the same τ parameter, NR-RDR-GMCC can realize an enhanced convergence rate and filtering accuracy by decreasing the values of *s* from 3.0 to 1.5.

**Figure 6:**
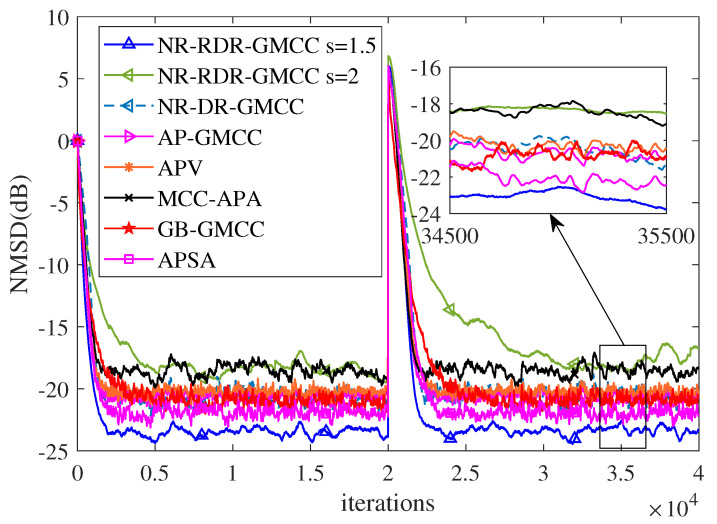
The NMSD curves of competing algorithms with $d_{\alpha }^{T}=\left[1.5,0,0,0.1\right]$.

Finally, in order to verify the effectiveness of the proposed NR-RDR-GMCC algorithm, some related algorithms, such as NR-DR-GMCC, GB-GMCC [19], affine projection GMCC (AP-GMCC) [20], AP sign algorithm (APSA) [29], AP Versoria (APV) [30] and MCC-APA [31], are considered in this part. For all algorithms, the smooth parameter or regularization parameter ϵ=0.001; for all GMCC based algorithms, s=1.5, τ=0.0001; other parameter settings are experimentally tested so that the algorithms have similar initial convergence rates. Table 2 lists the parameter setting. In addition, to further verify the advantages of the GMCC criterion, this experiment also considers the special case of s=2.0 in the NR-RDR-GMCC algorithm; in other words, the NR-RDR-GMCC algorithm reduces to an MCC-based NR-RDR algorithm. Furthermore, we change the unknown weight vector from $w^{o}$ to $-w^{o}$ at the middle iterations to simulate system mutation and explore the tracking performance of various algorithms. Figure 6 plots the corresponding NMSD curves and reveals that: (1) Compared to the GB-GMCC algorithm, the NR-RDR-GMCC (s=1.5) algorithm has a very obvious advantage in terms of filtering accuracy and convergence rate. In addition, irrespective of the mutation of system, in comparison with other algorithms, the NR-RDR-GMCC (s=1.5) algorithm achieves the comparable convergence rate and the best filtering accuracy; (2) In this simulation, the NR-RDR-GMCC (s=2.0) algorithm is inferior to the other algorithms in terms of convergence rate and steady-state misadjustment as shown in Table 2. That is to say, in some situations, to derive some robust adaptive filtering algorithms, GMCC is better than MCC.

### 4.2. Acoustic Echo Cancellation

**Figure 7:**
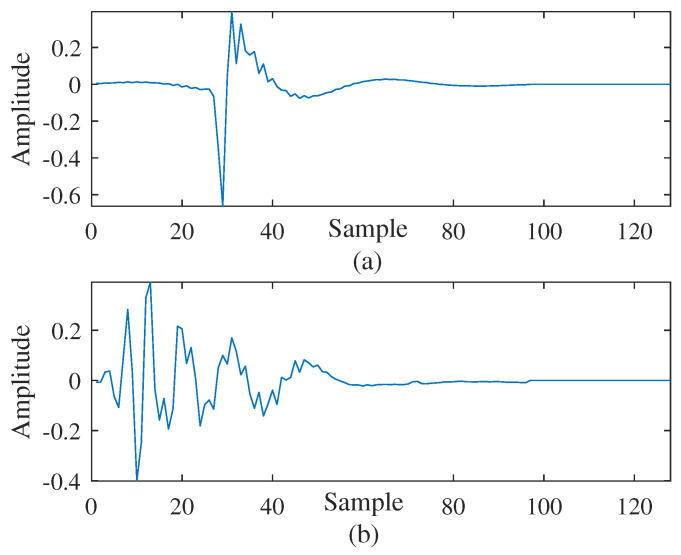
Two acoustic finite impulse responses with L=128: (**a**) $w_{1}^{o}$; (**b**) $w_{2}^{o}$.

**Figure 8:**
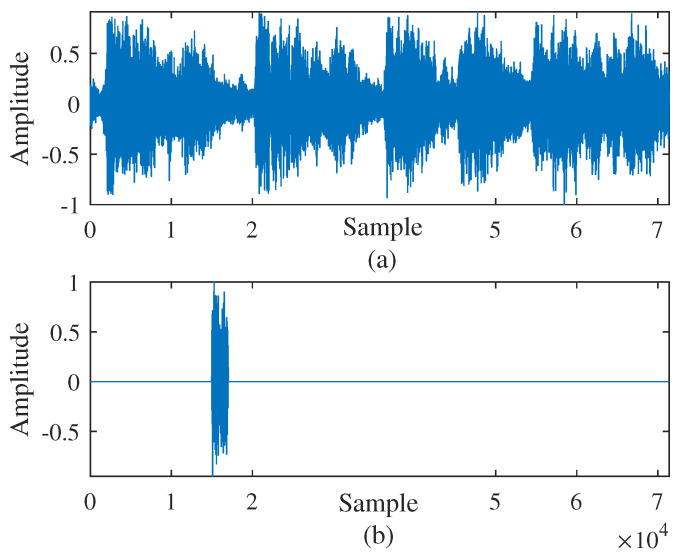
Speech signals: (**a**) Far-end; (**b**) Near-end.

To further compare the filtering behaviors of the mentioned algorithms, a typical application, i.e., acoustic echo cancellation (AEC), is also considered in this work. Under the condition of double-ended voice, in this part, two kinds of environmental noises are considered, namely: (1) the noise v(n) being impulsive and (2) the noise v(n) not being impulsive. In all trials, a Gaussian background noise with a signal-to-noise ratio of 60 dB was considered. The echo paths $w_{1}^{o}$ and $w_{2}^{o}$ [32] involved in this experiment and the voice input signal are shown in Figure 7 and Figure 8, respectively, where the signal sample rate was set to 8 kHz. To simulate the system mutation, the echo path was abruptly changed from $w_{1}^{o}$ to $w_{2}^{o}$ at the 7000-th iteration. Except where otherwise mentioned, for all algorithms, the data-reusing order or projection order is M=16, the regularization parameter or smooth parameter is ϵ=0.0001, and for GMCC-based algorithms the kernel factor is τ=0.001.

#### 4.2.1. Without Impulsive Noise

**Figure 9:**
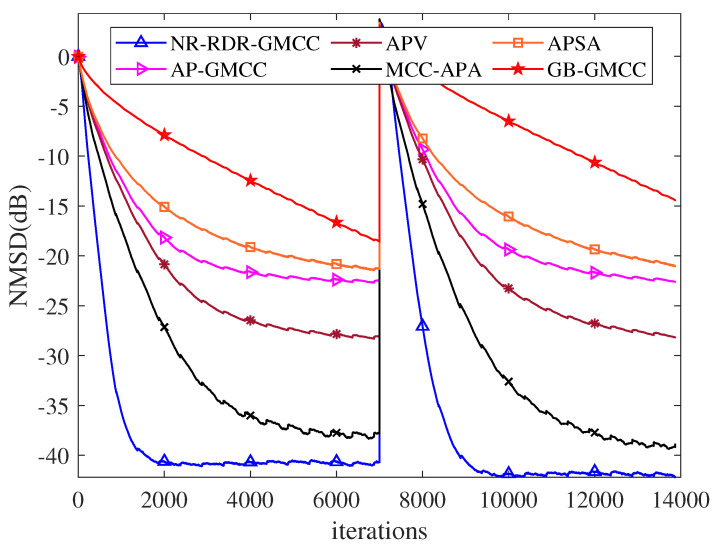
The NMSD curves of various algorithms in ACE without impulsive noise interference.

In order to compare the acoustic echo cancellation filtering performance of the NR-RDR-GMCC algorithm and other related algorithms in an environment without impulsive noise, based on various experimental tests, Table 3 lists the remaining parameters’ setting to realize a similar initial convergence rate. Figure 9 plots the corresponding NMSD curves. From this figure, we can see that: (1) Due to the interference of near-end voice, the NR-RDR-GMCC algorithm and other related algorithms have a slightly jitter phenomenon after convergence; (2) Without interference of impulsive noise, NR-RDR-GMCC is superior to other algorithms in terms of convergence rate and filtering accuracy before or after system mutation.

#### 4.2.2. With Impulsive Noise

**Figure 10:**
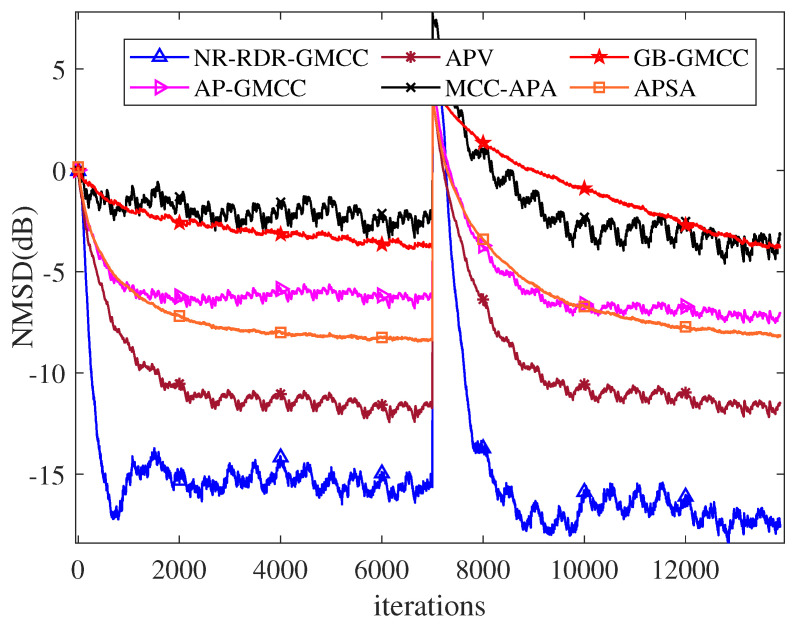
The NMSD curves of various algorithms in ACE under an impulsive noise environment.

In this part, we use the Bernoulli–Gaussian distribution model to obtain the impulsive noise, i.e., $v\left(n\right)=b\left(n\right)v_{g}\left(n\right)$, where b(n) is the Bernoulli process with the probability density model $P\left\{b(n)=1\right\}=Pr=0.001$. Before the system mutation, $v_{g}\left(n\right)$ is a zero-mean Gaussian noise with a variance of 151. After system mutation, the variance of $v_{g}\left(n\right)$ becomes 110. For all algorithms, Table 4 lists the remaining parameters’ setting to realize a similar initial convergence rate. The corresponding NMSD curves are plotted in Figure 10, which shows that, although some impulsive noises may have a negative effect on the filtering performance of the considered algorithms, our proposed NR-RDR-GMCC still achieves the smallest steady-state misadjustment before or after the mutation of the system.

## 5. Conclusions

The traditional GB-GMCC algorithm has a degradation of convergence performance when facing highly colored input signals. In this work, to overcome this issue, the NR-DR-GMCC algorithm was derived by using the data-reusing method and the Newton recursion method. In addition, to further avoid the influence of continuous large outliers in the output signal and extract more historical data, a cache window is considered to collect the latest *C* input vectors, and then *M* input vectors in the cache window are randomly selected to derive an enhanced version of NR-DR-GMCC, namely, NR-RDR-GMCC. From a mathematical point of view, when the cache window length *C* is equal to the data-reusing order *M*, NR-RDR-GMCC can be converted to the NR-DR-GMCC algorithm, and thus the former can be regarded as a good extension of the latter. The system identification simulation results show that the NR-RDR-GMCC algorithm has a better filtering accuracy and a faster convergence rate than the corresponding AP-type algorithm and GB-GMCC. Furthermore, in acoustic echo cancellation applications, the NR-RDR-GMCC algorithm has obvious advantages over other related algorithms in terms of steady-state misalignment.

## Figures and Tables

**Table 1:** Computational Complexity of Algorithms Per Iteration.

Algorithms	Multiplications	Additions
GB-GMCC	$MN_{exp}^{\tau ,s}+2L+1$	2ML+L
AP-GMCC	$MN_{exp}^{\tau ,s}+2ML+2L+1$	2ML+L
APSA	ML+2L+K	2ML+L
APV	M(2L+M+7)+1	2ML+L
MCC-APA	$O\left(L^{3}\right)+\left(M+2\right)L^{2}+N_{exp}^{\tau ,s}+2\left(M+1\right)L+2$	$\left(M+L\right)L^{2}+\left(2M-1\right)L+M-1$
NR-RDR-GMCC	$O\left(L^{3}\right)+\left(M+4\right)L^{2}+N_{exp}^{\tau ,s}+3\left(M+1\right)L+M$	$\left(M+1\right)L^{2}+\left(2M-1\right)L+M$

**Table 2:** Parameter Setting And Steady-State NMSD Results In System Identification.

Algorithms	η	*M*	*C*	Radius	Kernel Width	SS-NMSD (dB)	
				r	σ	$w^{o}$	$-w^{o}$
GB-GMCC	0.004	-	-	-	-	−20.9597	−20.8324
AP-GMCC	0.005	16	-	-	-	−20.7933	−20.6320
APSA	0.006	16	-	-	-	−22.1333	−22.0104
APV	0.005	16	-	10	-	−20.4574	−20.2859
MCC-APA	0.005	16	-	-	10	−19.1079	−18.7148
NR-DR-GMCC	0.002	16	16	-	-	−21.2425	−20.7102
NR-RDR-GMCC	0.005	16	512	-	-	−23.8190	−23.5575

**Table 3:** Parameter Setting and Steady-State NMSD Results in AEC without Impulsive Noise.

Algorithms	Parameters	SS-NMSD (dB)	
		$w_{1}^{o}$	$w_{2}^{o}$
GB-GMCC	η=0.05, s=1.7	−18.4309	−14.3984
AP-GMCC	η=0.10, s=1.7	−22.5124	−22.3412
APSA	η=0.06	−21.1401	−20.5691
APV	η=0.05	−28.0652	−27.7924
MCC-APA	η=0.50	−37.9557	−38.9585
NR-RDR-GMCC	η=0.10, C=256, s=1.7	−40.8235	−41.9047

**Table 4:** Parameter Setting and Steady-State NMSD Results in AEC under Impulsive Noise.

Algorithms	Parameters	SS-NMSD (dB)	
		$w_{1}^{o}$	$w_{2}^{o}$
GB-GMCC	η=0.15, s=1.6	−3.6680	−3.5960
AP-GMCC	η=0.20, s=1.6	−6.2958	−7.1930
APSA	η=0.30	−8.3213	−8.0550
APV	η=0.10	−11.7331	−11.6527
MCC-APA	η=0.10	−2.4777	−3.6380
NR-RDR-GMCC	η=0.10, C=256, s=1.6	−15.6412	−17.2785

## Data Availability

Not applicable.
